# Biases in the Explore–Exploit Tradeoff in Addictions: The Role of Avoidance of Uncertainty

**DOI:** 10.1038/npp.2015.208

**Published:** 2015-12-02

**Authors:** Laurel S Morris, Kwangyeol Baek, Prantik Kundu, Neil A Harrison, Michael J Frank, Valerie Voon

**Affiliations:** 1 Institute of Behavioural and Clinical Neuroscience, University of Cambridge, Cambridge, UK; 2 Department of Psychiatry, University of Cambridge, Addenbrooke's Hospital, Cambridge, UK; 3 Section on Functional Imaging Methods, National Institute of Mental Health, Bethesda, MD, USA; 4 Department of Psychiatry, Brighton and Sussex Medical School, Brighton, UK; 5 Department of Cognitive, Linguistic and Psychological Sciences, Brown Institute for Brain Science, Psychiatry and Human Behavior, Brown University, Providence, RI, USA; 6 Cambridgeshire and Peterborough NHS Foundation Trust, Cambridge, UK; 7 NIHR Cambridge Biomedical Research Centre, Cambridge, UK

## Abstract

We focus on exploratory decisions across disorders of compulsivity, a potential dimensional construct for the classification of mental disorders. Behaviors associated with the pathological use of alcohol or food, in alcohol use disorders (AUD) or binge-eating disorder (BED), suggest a disturbance in explore–exploit decision-making, whereby strategic exploratory decisions in an attempt to improve long-term outcomes may diminish in favor of more repetitive or exploitatory choices. We compare exploration *vs* exploitation across disorders of natural (obesity with and without BED) and drug rewards (AUD). We separately acquired resting state functional MRI data using a novel multi-echo planar imaging sequence and independent components analysis from healthy individuals to assess the neural correlates underlying exploration. Participants with AUD showed reduced exploratory behavior across gain and loss environments, leading to lower-yielding exploitatory choices. Obese subjects with and without BED did not differ from healthy volunteers but when compared with each other or to AUD subjects, BED had enhanced exploratory behaviors particularly in the loss domain. All subject groups had decreased exploration or greater uncertainty avoidance to losses compared with rewards. More exploratory decisions in the context of reward were associated with frontal polar and ventral striatal connectivity. For losses, exploration was associated with frontal polar and precuneus connectivity. We further implicate the relevance and dimensionality of constructs of compulsivity across disorders of both natural and drug rewards.

## INTRODUCTION

Tricky decisions arise almost daily, from the mundane, should I try something new for lunch today, to the more exotic, should I move to a different city? To navigate a dynamic world, individuals must adapt behavior and consider the trade-off between exploring an uncertain environment for the potential to improve beyond the status quo and exploiting known reward sources, in the hope of maintaining optimal decision-making. Behaviors associated with the pathological use of alcohol or food, in alcohol use disorders (AUD) or binge-eating disorder (BED), might suggest a disturbance in explore–exploit decision-making, whereby strategic exploratory decisions in attempt to improve long-term outcomes may diminish in favor of more repetitive or exploitatory choices. Here we aim to further characterize the trade-off between exploring the uncertain and exploiting the known in these groups.

Faced with an explore–exploit dilemma, one may initially randomly sample the environment and gradually reduce the probability of choosing each action with increasing outcome knowledge. However, descriptions using stochastic choice rules initially govern random exploration and do not take into account the amount of information that could be gained by sampling an unknown choice. Instead, choices may be directed by the amount gained by an exploratory choice (Badre *et al*, 2012; Frank *et al*, 2009; Dayan and Sejnowski, 1996). Within this framework, the level of certainty that a choice will engender a better than expected outcome, will influence exploratory choice. Using a temporal utility decision-making task, a recent study provided support for this assumption; the inclusion of an uncertainty term in computational modeling of trail-by-trial choices provided a superior description of exploratory choice (Frank *et al*, 2009). Thus, behavioral measures that are not accounted for by positive and negative prediction error updating can instead be explained as exploratory adjustments toward uncertainty (Badre *et al*, 2012; Cavanagh *et al*, 2012).

At a neural level, the frontopolar cortex (FPC) and intraparietal sulcus have been implicated in exploratory behaviors (Daw *et al*, 2006). With widespread cortical and subcortical anatomical and functional connectivity (Liu *et al*, 2013), the FPC sits at the top of a hierarchical behavioral control system, evaluating heterogeneous inputs for reward-related cognitive task integration in the pursuit of an advanced behavioral goal (Christoff and Gabrieli, 2000; Koechlin and Hyafil, 2007; Ramnani and Owen, 2004). Activity in FPC increases and decreases, with exploratory and exploitative decisions, respectively (Daw *et al*, 2006). In line with the role of uncertainty in driving exploratory choice, the lateral FPC has been shown to track the relative uncertainty of choices when exploratory choices are made and preferentially in those subjects who use an uncertainty-guided exploration strategy (Badre *et al*, 2012; Cavanagh *et al*, 2012). Striatal dopamine function, marked by functional polymorphisms in dopaminergic genes, has also been associated with exploitative decision-making by modulating learning from positive and negative prediction errors (Frank *et al*, 2009).

We focus on exploratory decisions across disorders of compulsivity, a potential dimensional construct for the classification of mental disorders in line with recent Research Domain Criteria strategies (Insel *et al*, 2010). Compulsivity can be described as repetitions of deleterious choices, which remain insensitive to changes in outcome contingencies and occur despite negative consequences (Robbins *et al*, 2012; Voon *et al*, 2014a). An outstanding question is to what extent exploratory choices are altered in disorders of compulsivity. We have recently shown that binge-eating, a compulsive pattern of food intake, presents similar behavioral characteristics to drug taking disorders including greater risk-taking for rewards (Voon *et al*, 2014c) and greater reliance on habitual learning strategies (Voon *et al*, 2014a). Binge-eating behavior provides a means of distinguishing crucial subtypes within obesity.

With a task previously shown to elicit uncertainty-driven exploratory decision-making behavior in humans (Badre *et al*, 2012; Frank *et al*, 2009; Cavanagh *et al*, 2012; Kayser *et al*, 2015), we compare on a behavioral level, exploration *vs* exploitation across disorders of natural (obesity with and without BED) and drug rewards (AUD). We expect a trans-pathological marker of reduced strategic uncertainty-driven exploratory behaviors compared with healthy volunteers (HV). We separately acquired resting state functional MRI (rsfMRI) data from healthy individuals to assess the neural correlates underlying exploration. We use a novel multi-echo planar imaging sequence and independent components analysis (ME-ICA) to separate blood oxygen level dependent (BOLD) from non-BOLD activity. This acquisition and analysis greatly enhances signal-to-noise ratios compared with traditional single-echo sequences thus allowing higher spatial resolution (Kundu *et al*, 2012). We focus on the connectivity of the FPC and hypothesize that connectivity with ventral striatum (reward valuation) and inferior parietal cortex (action implementation) is associated with exploratory behaviors in the context of reward. We secondarily assess exploration in the context of loss, expecting a similar network including FPC and inferior parietal cortex.

## MATERIALS AND METHODS

### Participants

We recruited HV from community and University-based advertisements in the East Anglia region, United Kingdom. The recruitment strategy for patient groups has been reported elsewhere (Voon *et al*, 2014b). For all patient groups primary diagnoses were confirmed by a psychiatrist using the Diagnostic and Statistical Manual of Mental Disorders, Version IV criteria for substance dependence or Research Diagnostic Criteria for BED (Association, 2000). Written informed consent was obtained and the study was approved by the University of Cambridge Research Ethics Committee. The same subjects completed the behavioral task outside of the scanner and underwent the rsfMRI scan. For further information see Supplementary Materials.

### Task

**Figure 1:**
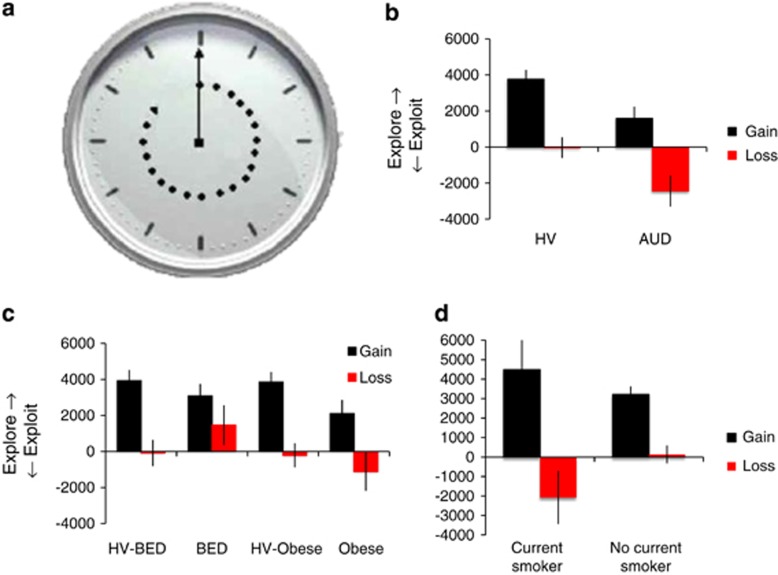
Exploratory behavior in disorders of natural and drug rewards. (a) Participants viewed a rotating clock and were instructed to stop the clock in order to win money or avoid losing money. The time at which the clock was stopped determined how much was won or lost. Exploratory choices are those that had not been previously sampled. (b) Explore–exploit index (represented in units of milliseconds per unit SD of the belief distributions) in alcohol use disorders (AUD) was lower than matched healthy volunteers (HV) (group effect; *p*=0.003). (c) Comparing obese subjects with binge-eating disorder (BED) and without (obese) with matched HV revealed no group differences. Comparing BED and obese revealed a group difference (*p*=0.04) when controlled for age, gender, and smoking status. (d) Comparing current smokers and non-smokers in HV revealed a group × valence interaction (*p*=0.03).

We used a task previously shown to elicit exploratory decision-making behavior in humans (Badre *et al*, 2012; Frank *et al*, 2009). Participants viewed a clock arm that rotates at 5 s per revolution (Figure 1). Participants were instructed to press the space bar before a full turn of the arm to win and were informed that the time at which the arm is stopped will determine how much money would be won. The outcome (£0–£200) was revealed for 1 s followed by an inter-trial interval of 300 ms. There were 40 trials per condition. An early key press did not affect the total time of the task and subjects were instructed to stop the clock at different times to maximize potential of winning.

In the previously described task, outcomes varied in probability and magnitude as a function of response times (RTs) such that expected value increased, decreased or remained constant with increasing RTs. In the current version of the task, only the conditions in which expected value was constant across the whole clock were used which engender most exploratory decisions, but with different frequencies and magnitudes. The increasing and decreasing conditions were replaced with a duplicate set of constant expected value conditions (both CEV and CEVReverse), but for which the outcomes were losses instead of gains. This allows us to assess whether participants use the same uncertainty-driven exploration strategy in the domain of losses, whether they are more averse to uncertainty in that case and whether compulsive individuals show any difference not only in exploration but in its modulation by valence. Exploratory choices are those made for clock arm positions (coarsely, fast *vs* slow portions of the clock) for which reward outcomes were more uncertain given previous samples (Badre *et al*, 2012). The relationship between the probability of winning or losing, the outcome magnitude and clock position was random hence was not associated with learning. Further task details and the computational model are reported in Supplementary Materials.

The model parameters were inspected for normality of distribution using Shapiro–Wilkes. For the exploration parameter, each group was compared with their own matched HV and assessed using mixed measures ANOVA with within-subject factor of valence (gain, loss) and between-subject factor of group. The BED and obese subjects were also directly compared. Data that were skewed (learning rates, *ρ*) were analyzed using Mann–Whitney *U*-tests.

### Resting State Functional MRI

We employed a novel ME-ICA in which BOLD signals were identified as independent components having linear TE-dependent signal change and non-BOLD signals were identified as TE-independent components (Kundu *et al*, 2012). Spatial smoothing was conducted with a Gaussian kernel (full width half maximum=6 mm). CONN-fMRI Functional Connectivity toolbox (Whitfield-Gabrieli and Nieto-Castanon, 2012) for Statistical Parametric Mapping SPM8 (http://www.fil.ion.ucl.ac.uk/spm/software/spm8/) was used for functional connectivity analysis. A strictly defined region of interest (ROI) for the FPC was used based on strong *a priori* hypotheses (Daw *et al*, 2006), to compute ROI-to-voxel connectivity maps. These maps were entered into second level correlation analysis with exploration behavioral measures, using cluster extent threshold correction calculated at 15 voxels at *p*<0.001 whole brain uncorrected, which corrects for multiple comparisons at *p*<0.05 assuming an individual-voxel Type I error of *p*=0.01 (Slotnick *et al*, 2003). Further details are reported in Supplementary Materials.

## RESULTS

The subject characteristics have been previously reported (Voon *et al*, 2014a,c; see Table 1). Thirty-two AUD subjects (weeks abstinent 16.62 (SD 16.72); years of dependence 13.67 (SD 9.40); units/day 27.28 (SD 13.95), on the following medications (acamprosate 2; disulfiram 1)), 31 obese with BED and 30 obese without BED were matched with their own age- and gender-matched HV (*N*=55 for each group). AUD and obese with BED had higher depression scores compared with HV. Obese with and without BED had higher body mass index (BMI) and obese with BED had higher Binge Eating Scale (BES) scores.

### Behavioral Characterization of Explore–Exploit Dilemma Across Disorders of Natural and Drug Rewards

The data from one healthy volunteer and one AUD were removed as they were >3 SD above the group mean. The exploration indices for gain and for loss were square root transformed.

Exploration indices were compared between gain and loss separately for each subject group using repeated measures ANOVA with smoking status as a covariate of no interest. Higher exploratory behaviors in the context of gain compared with loss was observed in HV (*F*(1.94)=511.77, *p*<0.001), AUD (*F*(1.29)=178.99, *p*<0.001), obese subjects (*F*(1.28)=109.17, *p*<0.001), and in BED (F(1.29)=72.10, *p*<0.001), supporting the interpretation that subjects are averse to uncertainty in the context of losses, possibly in the fear that their exploratory choices could yield yet worse outcomes.

In the AUD comparison with HV, there was a main valence effect (*F*(1.84)=36.00, *p*<0.001) and a group effect (*F*(1.84)=6.69, *p*=0.003) in which AUD subjects had lower exploration indices compared to HV with no interaction effect (*F*(1.84)=0.032, *p*=0.858) (Figure 1). With the addition of smoking status as a covariate of no interest, the group effect remained significant (*p*=0.035).

In the BED comparison with HV, there was a main valence effect (*F*(1.84)=4187.31, *p*<0.001) and no group (*F*(1.84)=0.46, *p*=0.499) or interaction effect (*F*(1.84)=1.50, *p*=0.224). In the obese comparison with HV, there was a main valence effect (*F*(1.83)=4105.23, *p*<0.001) and no group (*F*(1.83)=2.00, *p*=0.161) or interaction effect (*F*(1.83)=0.17, *p*=0.683). We then compared the BED and obese subjects which showed a trend towards a group difference (*F*(1.56)=3.47, *p*=0.068). With the addition of age, gender and smoking status as covariates of no interest, we show a main valence effect (*F*(1.56)=58.39, *p*<0.001) and main group effect (*F*(1.56)=4.60, *p*=0.037) in which obese subjects had lower exploration indices than BED. There was a trend towards an interaction between group × valence (*F*(1.56)=3.48, *p*=0.068). Posthoc analysis revealed significant differences between groups in the loss (*p*=0.041) but not gain condition (*p*=0.405).

We also compared AUD with BED subjects with age, gender, and smoking status as a covariate of no interest showing a main group effect (*F*(1.54)=9.19, *p*=0.004) in which AUD subjects were less exploratory than BED subjects; a main valence effect (*F*(1.54)=50.94, *p*<0.001); and a group × valence interaction (*F*(1.54)=8.60, *p*=0.005). Posthoc testing revealed significant group difference in the loss domain only (*p*=0.003) in which BED subjects were more exploratory compared with AUD subjects.

On an exploratory basis, we examined the influence of smoking status in HV. We identified 13 current smokers and 83 current non-smokers and compared these using mixed measures ANOVA. There was a main valence effect (*F*(1.94)=511.77, *p*<0.001) and a group × valence interaction (*F*(1.94)=5.02, *p*=0.027) in which smokers made more exploratory choices under gain and fewer exploratory choices under loss compared to non-smokers (Figure 1). There was no main group effect (*F*(1.94)=1.76, *p*=0.187).

The other parameter fits were also compared between AUD and HV and between obese subjects with and without BED. There were no differences in the other parameters (Table 2, Supplementary Figure S1). There were no correlations between the exploration indices and measures of alcohol severity, BMI, or BES.

### Frontal Polar Cortex Connectivity and Exploration

Of the participants that completed the task, 37 HV (20 male; mean age 35, SD 15; verbal IQ 115, and SD 11), underwent resting state fMRI with a multi-echo resting state sequence. This acquisition and an analysis greatly enhances signal-to-noise ratios compared with traditional techniques and provides enhanced spatial resolution based on robust physical priniciples (Kundu *et al*, 2012). The explore/exploit task was tested out of the scanner. The FPC was carefully defined and used as a seed. Connectivity was quantified by calculating Pearson correlations coefficients between activity within the seed and the whole brain, producing seed-to-voxel whole-brain connectivity maps. These maps were then correlated with the behavioral measure of exploration. Age was included as a covariate of no interest.

**Figure 2:**
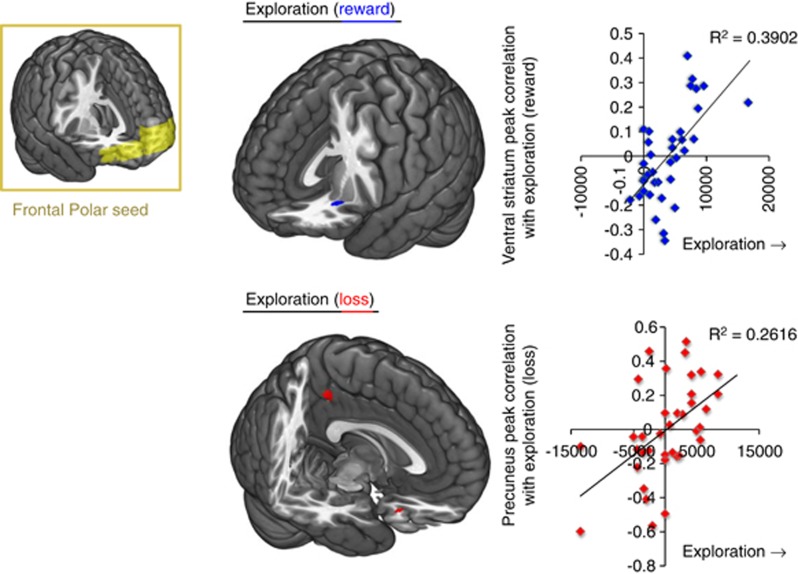
Neural correlates of exploratory decisions in healthy volunteers (HV). Left, frontal polar cortex (FPC) seed. Seed-to-whole brain connectivity maps were correlated with exploratory behaviors in the context of reward and loss. Middle, regions whose functional connectivity with FPC correlated with exploratory behavior. Right, parameter estimates extracted for illustrated peak coordinates are correlated with the exploratory behavior.

Cluster-extent threshold analysis (calculated at 15 voxels at *p*<0.001 whole-brain uncorrected, correcting for multiple comparisons at *p*<0.05 assuming an individual-voxel Type I error of *p*=0.01 (Slotnick *et al*, 2003)) revealed that exploration in the context of reward was positively correlated with FPC and ventral striatal connectivity (peak coordinates *x y z*=−22, 21, −10 mm; cluster size=32; *Z*=4.38, Figure 2). In the context of loss, greater exploration was positively correlated with greater FPC and precuneus connectivity (peak *x y z*=−1, −41, 42 mm; cluster size=24; *Z*=3.61).

**Figure 3:**
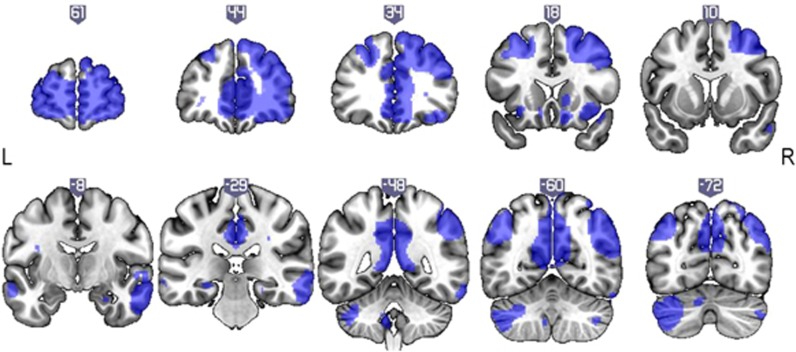
Frontal polar cortex connectivity. A frontal polar cortex (FPC) seed was correlated with the whole brain to produce seed-to-voxel functional connectivity maps. The connectivity map is displayed at *p*=0.001 uncorrected for illustration.

Finally we map connectivity of the FPC to the whole brain. At whole-brain FWE *p*<0.05 we find that FPC is functionally connected with a network including dorsolateral prefrontal cortex, precuneus, inferior parietal and subcortically, and the ventral striatum (Figure 3, Table 3).

## DISCUSSION

We employed a choice task previously used to demonstrate strategic exploratory decision-making behavior in healthy humans (Badre *et al*, 2012; Frank *et al*, 2009). All groups show a conserved effect of valence such that exploration was higher in the reward domain compared with the loss domain. Indeed, in the loss domains, subjects showed a consistently negative exploration parameter, meaning that they were averse to uncertainty when there was some prospect of losing even more. These findings potentially reflect the asymmetrical influence of gains and losses on choice behavior (Kahneman and Tversky, 1979) imposed by the strength of loss aversion as a consistent mediator of choice (Tom *et al*, 2007).

Exploratory behavior in subjects with AUD was reduced across gain and loss environments, in favor of more repetitive or exploitative choices. Obese subjects with and without BED did not differ from HV in their exploratory choices. However, when compared with each other, there was greater exploratory behaviors in BED subjects compared with those without BED. There was a trend toward a group × valence interaction driven by greater exploratory behaviors to losses in BED subjects compared with those without BED. Similarly, BED subjects had greater exploratory behaviors particularly to losses compared with AUD. Furthermore, we investigated the influence of smoking in HV on a pilot basis: current smokers showed an enhancement of the influence of valence with greater exploration to gain outcomes and less exploration to loss outcomes compared with non-smokers. Exploratory behavior in HV was associated with an underlying network including FPC and ventral striatal connectivity in the context of reward and FPC and precuneus for losses.

Compared with HV, AUD subjects had restricted exploratory behaviors and were more likely to avoid uncertainty across both gain and loss stimulus-outcomes in a task that is independent of learning. AUD subject have been shown to have abnormalities in decision making under ambiguity or uncertainty as measured using the Iowa Gambling Task (Goudriaan *et al*, 2005; Bechara *et al*, 2001). Our findings extend these results to suggest either intolerance/avoidance of uncertainty, or a reduced tendency to use a controlled strategy that searches for uncertain outcomes so as to maximize information gain. The current findings of reduced exploration in an unknown environment dovetail with findings suggesting that the effects of alcohol are selective for uncertainty-related anxiety rather than certainty-related fear (Hefner and Curtin, 2012), the former being hypothesized to drive the negative-reinforcement cycle of alcohol use (Edwards and Koob, 2010). An alternate explanation may be that changes in outcome sensitivity, rather than uncertainty avoidance, may engender reluctance to explore. However, decreased sensitivity to outcome may be more likely to manifest as greater exploration to sample further stimulus-outcome contingencies. Although we do not explicitly measure the role of novelty, decreased exploration may relate to the possible presence of novel environments. Ethanol withdrawal in rodents indeed causes reduced exploration of brightly lit chambers (Hascoet *et al*, 2001).

Furthermore, like HV, AUD subjects had decreased exploratory behaviors to losses compared with gains suggesting sensitivity to their differential influences. Current smokers also have an enhancement of this differential effect of valence with greater exploratory behaviors to gains and the opposite to losses relative to non-smokers. The enhancement in exploration for gains is in line with enhanced reward sensitivity related to nicotine use (Rose *et al*, 2013). This finding invites the suggestion that participants who are more likely to explore the potential hedonic benefits of smoking are those that become smokers. The findings in the loss domain suggest a potential role for enhanced loss aversion in smokers with greater avoidance of uncertainty in a loss context, perhaps facilitating sustained smoking in the presence of perceived small losses associated with immediate health consequences, rather than explore alternative strategies that would require giving up smoking for potentially other (eg social) losses. Although losses in the form of social and health cost are difficult to model, the secondary reinforcer of money can act as a proxy. These findings in AUD and smokers may be consistent with the negative reinforcement model of addiction (Koob, 2013; Koob and Le Moal, 2005) whereby a negative context may drive exploitative repetitive behaviors to avoid losses. Reduced exploration, or more repetitive choices, in the face of losses is consistent with theories that neuroadaptive systems driving aversive states lead to repetitive drug-seeking behaviors (Edwards and Koob, 2010). Indeed, negative affect in smokers is associated with craving severity (Robinson *et al*, 2011). Together with the current findings, this may explain how particular environmental influences (ie negative outcomes in the form of financial, social, or health losses) may facilitate the repetition of behaviors with certain, known outcomes, such as pathological drinking and smoking behaviors. Although these findings are intriguing, we caution that the findings in smokers are preliminary as the sample size of current users is small, and we cannot rule out an impact of nicotine etc. on exploration rather than the other direction of causality.

That subjects display reduced exploration for losses contrasts with the observation of enhanced ambiguity seeking in the face of losses in healthy humans (Ho *et al*, 2002; Chakravarty and Roy, 2009). However, this discrepancy is also similar to the observation of ambiguity aversion in the face of gains, despite exploration toward uncertain options in that case. The main difference is that in a learning task, choosing an ambiguous option can serve to reduce subsequent ambiguity, ie exploration drives learning. In the case of losses, it is thus perhaps surprising that subjects do not seek uncertain options to reduce subsequent ambiguity. In addition, the current study deals with explicit and experienced uncertainty rather than hypothetical ambiguity. The effect of valence on risky choice has been shown to be reversed when choices are either experience or description-based, with the former reducing risk-seeking for losses (Ludvig and Spetch, 2011) consistent with our findings. Furthermore, there may be at least two strategies for approaching an explore–exploit dilemma: choice biased toward information seeking; and random exploratory decisions involving chance (Wilson *et al*, 2014) and perhaps subjects adopted a strategy to simply increase random choices in the case of losses rather than rely on uncertainty.

Our findings show decreased exploration in obese subjects without BED as compared with BED suggesting differences as a function of greater avoidance of uncertainty. BED subjects appear to be more biased toward exploratory behavior but particularly in the context of losses and not to gains, that is, the opposite profile from smokers. These findings are similarly evident in the comparison of AUD and BED subjects in which BED have greater exploratory behaviors and particularly in the loss domain. This dissociation of valence coincides with previous work showing that BED subjects demonstrate greater risk taking for high probability losses only (Voon *et al*, 2014c) possibly suggesting less of an influence of loss aversion. These findings suggest differences between AUD and BED subjects particularly in the loss domain. Whether the distinct rewards of choice (natural or drug) are responsible for causing increased or decreased exploration in the face of loss or whether they are a product of an inherent attraction or aversion to exploration, remains a question for future studies. The suggestion that neuroadaptive negative reinforcement systems are initiated or propagated by excessive reward system activation (Koob, 2013), may explain the current finding of heightened sensitivity to losses in smokers and individuals with AUD, but not in BED, whereby nicotine and alcohol hijack the reward system to a greater degree than food. Moreover, we note that the negative consequences of binge eating on weight gain are far more immediate than those of smoking, which are perceived to be delayed and subject to potential quitting.

Our findings further highlight a role for an intrinsic network of FPC connectivity in exploration biases. The FPC sits at the outermost periphery of the hierarchical prefrontal control regions (Christoff and Gabrieli, 2000; Koechlin and Hyafil, 2007), being well poised to mediate higher level strategic switches rather than behavioral sequence control. Accumulating evidence suggests that through interactions with social/emotional network (orbitofrontal cortex, amygdala), cognitive network (dorsolateral prefrontal cortex) and default mode network (precuneus, anterior cingulate cortex; Liu *et al*, 2013), the FPC orchestrates more flexible and self-relevant behavioral control in the pursuit of optimal decision-making (Koechlin and Hyafil, 2007). We show that FPC and ventral striatal connectivity is associated with exploration in the context of a rewarding environment. This coincides with the notion that the FPC coordinates voluntary and adaptive switching based on uncertainty and expected value (Badre *et al*, 2012; Daw *et al*, 2006). Exploration may depend on the probability that an explored choice will provide a better outcome than expected based on previous experiences (a positive prediction error; Frank *et al*, 2009). It is thus possible that the FPC-VS connectivity implies a reward value assignment to the potential for exploring. This would not be expected in the context of losses because the value of exploring is only to reduce loss values rather than provide a positive outcome.

We also show that FPC and precuneus connectivity positively correlates with exploration in the loss domain. Although the precuneus has been traditionally associated with integration of visuo-spatial imagery (Selemon and Goldman-Rakic, 1988), converging evidence suggests a role in integration of external and self-relevant information (Cavanna and Trimble, 2006). Furthermore, goal-directed hand movements (Karnath and Perenin, 2005) and voluntary attentional shifts between targets even in the absence of an overt motor response (Culham *et al*, 1998), are mediated by the precuneus. Functional links between FPC and the default mode network (Liu *et al*, 2013) support its role in processing internal rather than external generation of information (Christoff and Gabrieli, 2000) to guide future-focused (Okuda *et al*, 2003) decision-making. The current findings suggest that although assignment of perceived agency to actions and encoding and organizing of intentions is mediated by the precuneus, it may interact with the FPC (Liu *et al*, 2013) which in turn processes internally-generated goals for behavioral control (Ramnani and Owen, 2004; Okuda *et al*, 2003). Further evidence of the role of the precuneus in exploratory choices comes from studies of foraging behavior. Humans may alternate between economic decisions and choices governed by sequential ‘engage or search elsewhere' foraging choices (Kolling *et al*, 2012). Foraging choices (compared with decisions between two options) have been associated with activations in the precuneus extending to posterior cingulate cortex (PCC; Kolling *et al*, 2012) and PCC seems to be sensitive to risker compared with safer choices (Kolling *et al*, 2014). That this region is associated with risker choices suggests why it may be associated with exploratory choices losses rather than rewards.

Although recent evidence implicates both FPC and inferior parietal cortex in exploratory choices (Daw *et al*, 2006; Boorman *et al*, 2009), we did not find significant correlations for inferior parietal cortex. In a previous study, activity in both FPC and the inferior parietal sulcus correlated with the ratio between an unchosen and chosen action probability, or the relative unchosen probability (Boorman *et al*, 2009). However, the inferior parietal sulcus was only recruited when a switch in choice occurred (Boorman *et al*, 2009). Therefore, the FPC seems to track information accumulation relevant to switching to an alternate choice—here to reduce uncertainty—but engages the parietal cortex immediately before switching, which implements the switch itself. In line with this hypothesis, a recent study examining negative outcomes implicated the inferior parietal cortex in encoding actions and outcome objects but a more medial region, similar to that implicated in the current study, in encoding the action × object interaction reflecting the appropriate or inappropriate action (Morrison *et al*, 2013).

Our findings suggest biases in exploratory behaviors in the context of an uncertain environment across the misuse of drug and natural rewards. We also highlight the conserved effect of valence on exploration across groups with enhanced uncertainty avoidance to losses possibly reflecting an interaction with underlying loss aversion tendencies. Although we do not currently examine the neural correlates of exploration in the pathological groups, we build upon the understanding of the role of the FPC in guiding higher order and flexible decision-making, illustrating the possible means through which it coordinates behavioral processes in HV. Together, the findings further the characterization of overlapping disorders of natural and drug rewards by maintaining the use of dimensional facets of compulsivity.

## FUNDING AND DISCLOSURE

The study was funded by the Wellcome Trust Fellowship grant for VV (093705/Z/10/Z) and Cambridge NIHR Biomedical Research Centre. MJF is funded by NIMH and NSF grants, and is consultant for Hoffmann–LaRoche pharmaceuticals. The remaining authors declare no conflict of interest.

## Figures and Tables

**Table 1:** Subject Characteristics

	**AUD**	**HV–AUD**	**T P**	**Obese BED**	**HV**	**T P**	**Obese control**	**HV**	**T P**
*N*	32	55		31	55		30	55	
Age	41.29 (11.38)	42.15 (11.91)	0.330 0.742	42.51 (8.92)	43.18 (10.31)	0.303 0.762	44.06 (9.70)	42.94 (9.57)	0.513 0.609
Males (*N*)	19	32		14	25		19	34	
IQ	114.11 (6.72)	115.49 (6.33)	0.959 0.340	115.95 (6.67)	114.52 (6.73)	0.949 0.345	115.18 (6.45)	114.71 (6.83)	0.309 0.758
BDI	11.92 (9.33)	5.24 (5.75)	4.147 <0.001	13.49 (7.13)	5.48 (5.69)	5.706 <0.001	6.96 (5.92)	5.21 (5.13)	1.422 0.159
BMI				34.68 (5.49)	22.18 (2.59)	14.334 <0.001	32.72 (3.41)	23.14 (2.88)	13.72 <0.001
BES				24.70 (7.56)	6.57 (6.92)	11.282 <0.001	8.67 (7.08)	6.98 (7.14)	1.045 0.299

Abbreviations: AUD, alcohol use disorder; BDI, Beck Depression Inventory; BED, binge eating disorder; BES, Binge Eating Scale; BMI, body mass index; HV, healthy volunteer; *N*, number of participants.

**Table 2:** Best Fitting Model Parameters and Model Fit

	***K***	***λ***	***α***_**Gain**_	***α***_**Loss**_	***ρ***	***ν***	**SSE**
HV (AUD)	1016.95 (356.61)	0.35 (0.09)	0.15 (0.33)	0.08 (0.20)	257.66 (416.14)	0.35 (0.09)	5646.64 (742.33)
AUD	1007.80 (385.15)	0.34 (0.11)	0.11 (0.22)	0.02 (0.03)	362.32 (646.31)	0.38 (0.10)	5441.61 (585.11)
*t*	0.12	0.80			−0.93		1.399
*p*-value	0.907	0.426	0.496^a^	0.128^a^	0.354	0.271^a^	0.166
Obese	1227.34 (376.54)	0.30 (0.10)	0.19 (0.37)	0.13 (0.25)	545.12 (729.82)	0.36 (0.10)	5424.07 (780.23)
BED	1064.25 (463.31)	0.33 (0.10)	0.15 (0.33)	0.12 (0.33)	271.84 (486.92)	0.39 (0.13)	5420.22 (1058.00)
*t*	−1.55	1.09			−1.78		−0.02
*p*-value	0.127	0.280	0.476^a^	0.133^a^	0.080	0.130^a^	0.987

Abbreviations: AUD, alcohol use disorder; BED, binge eating disorder; HV, healthy volunteers; SSE, summed square of residuals (model fit).

a Mann–Whitney test.

**Table 3:** Statistics of FPC and Whole Brain Connectivity

	***p*** **(FWE-corr)**	**Cluster size**	***Z***	***x***	***y***	***z***
Frontal cortex (including medial PFC and anterior cingulate)	<0.001	10095	>8	−29	66	7
			>8	34	63	−3
			>8	27	66	2
Parietal cortex	<0.001	1205	>8	48	−58	49
	<0.001	896	>8	−41	−62	53
			>8	−43	−60	42
Cerebellum	<0.001	1280	>8	−43	−67	−38
			7.49	−13	−81	−28
			6.32	−22	−83	−26
Dorsolateral PFC	<0.001	1060	>8	−45	26	44
			>8	−24	31	53
			6.78	−34	17	56
Posterior cingulate (including precuneus)	<0.001	1482	>8	−1	−41	35
			7.77	1	−30	39
			7.08	−1	−69	42
Temporal cortex	<0.001	483	7.63	69	−16	−14
			7.61	66	−34	−12
	<0.001	149	6.26	−66	−39	−17
	0.018	2	4.9	−62	−20	−7
Anterior insula	<0.001	23	5.36	41	19	−10
			4.96	34	24	−10
Ventral striatum	0.013	3	5.04	−10	17	0
	0.018	2	4.97	13	17	4

Abbreviations: PFC, prefrontal cortex; *p*(FWE-corr), whole brain (*p*<0.05) family wise error corrected *p*-value; xyz, peak voxel coordinates; *Z*, *Z*-score.
