# Supplementary figures and images for: Biases in the Explore–Exploit Tradeoff in Addictions: The Role of Avoidance of Uncertainty
Source: Neuropsychopharmacology. 2015 Dec 2;41(4):940–8. doi: 10.1038/npp.2015.208 (PMC4650253; doi:10.1038/npp.2015.208)

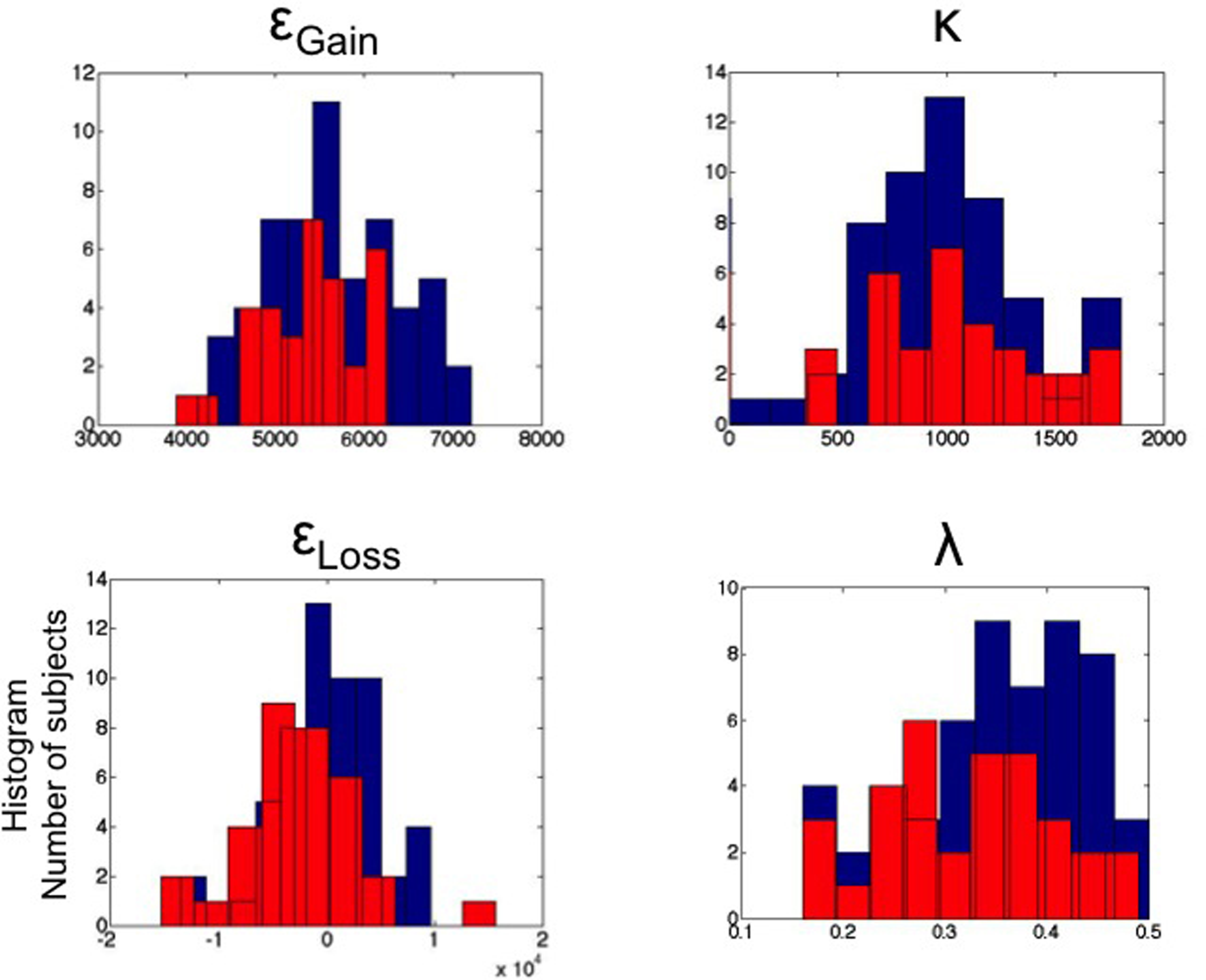

Supplement: Supplementary Figure S1 [file npp2015208x1.tif]
