# Supplementary material for: Biases in the Explore–Exploit Tradeoff in Addictions: The Role of Avoidance of Uncertainty
Source: Neuropsychopharmacology. 2015 Dec 2;41(4):940–8. doi: 10.1038/npp.2015.208 (PMC4650253; doi:10.1038/npp.2015.208)
Supplement: Supplementary Figure Legends [file npp2015208x2.docx]

Figure S1. Histogram plots of parameter fits in alcohol use disorders

Histograms are plotted for alcohol use disorders (red) and healthy volunteers (blue) for exploration indices for gain (top left) and loss (bottom left), κ (top right) and λ (bottom right).
