# Supplementary material for: Biases in the Explore–Exploit Tradeoff in Addictions: The Role of Avoidance of Uncertainty
Source: Neuropsychopharmacology. 2015 Dec 2;41(4):940–8. doi: 10.1038/npp.2015.208 (PMC4650253; doi:10.1038/npp.2015.208)
Supplement: Supplementary Information [file npp2015208x3.docx]

Supplemental Materials

Participants

Subjects >18 years old were included. HV, AUD and obese subjects with and without BED, were excluded if they had a current major depression or other major psychiatric disorder including substance addiction (except nicotine), major medical illness or were on psychotropic medications except acamprosate and disulfiram. Healthy volunteers were separately compared to the patient groups. Obese subjects completed the Binge Eating Scale (BES)^1^. Participants completed the Beck Depression Inventory to assess depressive symptoms. Psychiatric disorders were screened with the Mini International Neuropsychiatric Interview^2^. The National Adult Reading Test was used to assess IQ. Participants were compensated for their time and paid an additional amount depending on their performance.

Task

In the previously described task, outcomes varied in probability and magnitude as a function of response times (RT) such that expected value increased, decreased or remained constant with increasing response times. Flexible learning of expected values would produce faster RT’s (go learning) in the decreasing condition and slower RT’s (no-go learning) in the increasing condition. Thus, RT’s were adjusted in a continuous manner. A constant condition acted as a baseline for RT. A fourth condition was also included with increasing reward probability and decreasing magnitude, having the same expected values as the constant condition thus providing a means of examining biases towards probability or magnitude, as well as towards learning from negative outcomes. In the current version of the task, only the conditions in which expected value was constant across the whole clock were used which engender most exploratory decisions, but with different frequencies and magnitudes. The increasing and decreasing conditions were replaced with a set of certain conditions for which the outcomes were losses instead of gains. Subjects completed two blocks of either winning or losing money in random order.

Computational analysis

The following is adapted from Frank et al. (2009), Badre et al. (2012) and Kayser et al (2015), updated to include separate mechanisms for exploring based on gains and losses (the original task included gains only). For details about fitting and other motivations, please see the above references. The computational model presumes that trial-by-trial shifts in reaction time (RT) change as a function of reward prediction error and that strategic exploratory behavior can be modeled as a function of reward uncertainty.

The model assumes as with all reinforcement learning models that the expected value V(t) of a reward or loss for a given trial t is updated as follows:

V(t+1) = V(t) + αδ(t)

in which α is a learning rate indicating the extent to which values are updated and δ is the reward prediction error, or the presumed phasic dopaminergic signal representing the difference between the actual reward outcome versus the prior expected value:

δ(t) = Rew(t) – V(t).

To respond adaptively in the task, we assume the learner tracks whether they are more likely to obtain a better outcome than usual responding fast or slow. To do this, the model tracks the Bayesian belief distributions about the probability of obtaining a positive reward prediction error for fast or slow responses, and then adjusts RTs as a function of the differences between the means of these belief distributions. Thus the exploitation term of the model is as follows:

Exploitation (t) = ρ * [ μ _slow_ (t) – μ _fast_ (t) ]

where the magnitude of Exploitation at time *t* is equal to the difference between the means *μ* of the fast and slow distributions at that time, weighted by *ρ* fit to each subject’s data. This component of the model predicts that subjects will increase RTs if the average reward is greater for slow than fast responses, and vice versa. However, because the subject does not know in advance whether they will be likely to do better for responding fast or slow (and in fact in this version of the task the overall expected value is constant), they need to explore to obtain sufficient statistics about fast and slow responses. The explore term indexes the degree to which subjects are likely to guide exploration toward the more uncertain of these two (fast/slow) distributions to gather information about the reward space. Belief distributions that are wide (i.e. large standard deviations) are more uncertain than those which have been sampled more often, even for the same mean, In other words, subjects with larger explore parameters will tend to use their lack of knowledge about a part of the reward space to explore it further, thereby reducing the associated uncertainty.

Exploration (t) = ε * [σ_slow_ (t) - σ_fast_ (t)]

where the magnitude of Exploration at time *t* is equal to the difference between the standard deviations *σ* of the fast and slow distributions at that time, weighted by *ε* fit to each subject’s data. *ε* therefore represents, in units of milliseconds per unit standard deviation of the belief distributions, how strongly

the differences between the standard deviations are used to exploratory RT swings. This component of the model predicts that subjects will increase response times (RTs) if the outcome statistics are more uncertain for slow than fast responses, and vice versa

For completeness, we also model the more incremental and biased effects of reward prediction errors on learning in the downstream striatum, whereby positive prediction errors act to encourage speeded responding via the “Go” pathway and negative prediction errors act to slow responding via the “NoGo” pathway, thereby incorporating separate learning rates. Evidence for speeding and slowing in the task is separately tracked:

Go(*t*) = Go(*t*-1) + *α*_G_ δ _+_(*t*-1)

NoGo(*t*) = NoGo(*t*-1) + *α*_N_δ _−_(*t*-1)

where *α*_G_ and *α*_N_ are the learning rates; asymmetries in these learning rates have been previously linked to variations in striatal dopamine which have explained differences in the ability to speed up or slow down to maximize expected value ^3,4^. However, we note that this particular task design, the expected value is constant across the trial for all conditions, and thus not optimized to test differences due to these striatal components. Moreover, this incremental learning process does not consider when it is best to respond in a strategic manner, and in fact, it is not adaptive in environments where slow responses yield higher rewards (in which case positive and negative RPE's will lead to maladaptive RT adjustments)

The full model contributing to changes in RT is thus as follows:

RT(t) = K + λsticky(t)+ ρ[μ_slow_(s,t) - μ_fast_(s,t)] + Explore(s,t) + Go(s,t) – NoGo(s,t)

Where the free parameter K is the baseline response speed regardless of reward, λ is the autocorrelation between the current and previous RT’s (independent of reward). Consistent with prior work (Badre et al, 2012; Kayser et al, 2015) in this autocorrelation we allow for previous RTs beyond just the last by including a “sticky choice” parameter. Here, sticky(t) = RT(t-1) + *d**sticky(t-1), with 0<*d*<1 is a decay parameter influencing the degree to which prior RTs continue to affect current RTs, with exponentially decaying history. Incorporating this parameter allows us to better estimate the exploration component based on uncertainty *per se* so as to predict RT swings despite the overall tendency to stick with the same response.

Resting state functional MRI data acquisition and analysis

Data was acquired with a Siemens 3T Tim Trio scanner using a 32-channel head coil at the Wolfson Brain Imaging Centre at the University of Cambridge. A T1-weighted magnetization prepared rapid gradient echo (MPRAGE) sequence (176 x 240 FOV; 1-mm in-plane resolution; inversion time (TI), 1100ms) was used for acquisition of anatomical images. Healthy volunteer participants underwent an rsfMRI scan of 10 minutes with eyes open. These images were acquired with a multi-echo echo planar imaging (ME EPI) sequence with online reconstruction (repetition time (TR), 2.47s; flip angle, 78°; matrix size 64 x 64; in-plane resolution, 3.75mm; field of view (FOV), 240mm; 32 oblique slices, alternating slice acquisition slice thickness 3.75mm with 10% gap; iPAT factor, 3; bandwidth (BW) = 1,698 Hz/pixel; TE = 12, 28, 44 and 60 ms).

We used multi-echo independent component analysis (ME-ICA v2.5 beta6; [http://afni.nimh.nih.gov](http://afni.nimh.nih.gov/pub/dist/doc/program_help/@Install_MEICA_Demo.html)) for denoising of the multi-echo rsfMRI data. With this, multi-echo rsfMRI data is deconstructed into independent components using FastICA followed by component categorization as BOLD or non-BOLD based on the pseudo-*F*-statistics of Kappa and Rho values, respectively. BOLD percent signal change is linearly dependent on echo time (TE), a characteristic of the T2* decay. TE dependence of BOLD signal is measured using Kappa whereas non-BOLD components are identified by TE independence measured by Rho^5^. Non-BOLD components are removed (by projection), robustly denoising data for motion, physiological and scanner artefacts based on physical principles^6^. Each individual’s denoised echo planar images are coregistered to each individual’s MPRAGE and normalized to the Montreal Neurological Institute template.

CONN-fMRI Functional Connectivity toolbox^7^ for Statistical Parametric Mapping SPM8 (<http://www.fil.ion.ucl.ac.uk/spm/software/spm8/>) was used for functional connectivity analysis. Within the toolbox, voxel time course data were temporally band-pass filtered (0.008 < f < 0.09 Hz) and each individual’s anatomical scan was segmented into grey matter, white matter and cerebrospinal fluid (CSF) with significant principle components of white matter and CSF signals being removed. A strictly defined region of interest (ROI) for the frontal polar cortex was used based on strong *a priori* hypotheses^8^, to compute ROI-to-voxel connectivity maps. For the ROI we used Brodmann area 10 from WFU PickAtlas and manually restricted the ROI posteriorly at the boundary of the anterior coronal place where the three frontal gyri are present ^9-11^, and dorsally by the dorsal cytoarchitectonic extent of area 10p described by ^10^. Seed-to-whole brain functional connectivity maps were examined for whole brain connectivity of frontal polar cortex and then entered into second level correlation analysis with exploration behavioural measures described as covariates. Whole brain voxel-wise correlations were performed using cluster extent threshold correction. The cluster extent correction was calculated at 15 voxels at p<0.001 whole brain uncorrected, which corrects for multiple comparisons at p<0.05 assuming an individual-voxel Type I error of p=0.01^12^.

References

1 Gormally, J., Black, S., Daston, S. & Rardin, D. The assessment of binge eating severity among obese persons. *Addictive behaviors* **7**, 47-55 (1982).

2 Sheehan, D. V. *et al.* The Mini-International Neuropsychiatric Interview (M.I.N.I.): the development and validation of a structured diagnostic psychiatric interview for DSM-IV and ICD-10. *The Journal of clinical psychiatry* **59 Suppl 20**, 22-33;quiz 34-57 (1998).

3 Frank, M. J., Doll, B. B., Oas-Terpstra, J. & Moreno, F. Prefrontal and striatal dopaminergic genes predict individual differences in exploration and exploitation. *Nature neuroscience* **12**, 1062-1068, doi:10.1038/nn.2342 (2009).

4 Moustafa, A. A., Cohen, M. X., Sherman, S. J. & Frank, M. J. A role for dopamine in temporal decision making and reward maximization in parkinsonism. *J Neurosci* **28**, 12294-12304, doi:10.1523/JNEUROSCI.3116-08.2008 (2008).

5 Kundu, P., Inati, S. J., Evans, J. W., Luh, W. M. & Bandettini, P. A. Differentiating BOLD and non-BOLD signals in fMRI time series using multi-echo EPI. *NeuroImage* **60**, 1759-1770, doi:10.1016/j.neuroimage.2011.12.028 (2012).

6 Kundu, P. *et al.* Integrated strategy for improving functional connectivity mapping using multiecho fMRI. *Proceedings of the National Academy of Sciences of the United States of America* **110**, 16187-16192, doi:Doi 10.1073/Pnas.1301725110 (2013).

7 Whitfield-Gabrieli, S. & Nieto-Castanon, A. Conn: a functional connectivity toolbox for correlated and anticorrelated brain networks. *Brain connectivity* **2**, 125-141, doi:10.1089/brain.2012.0073 (2012).

8 Daw, N. D., O'Doherty, J. P., Dayan, P., Seymour, B. & Dolan, R. J. Cortical substrates for exploratory decisions in humans. *Nature* **441**, 876-879, doi:10.1038/nature04766 (2006).

9 Ramnani, N. & Owen, A. M. Anterior prefrontal cortex: insights into function from anatomy and neuroimaging. *Nature reviews. Neuroscience* **5**, 184-194, doi:10.1038/nrn1343 (2004).

10 Ongur, D., Ferry, A. T. & Price, J. L. Architectonic subdivision of the human orbital and medial prefrontal cortex. *The Journal of comparative neurology* **460**, 425-449, doi:10.1002/cne.10609 (2003).

11 Ongur, D. & Price, J. L. The organization of networks within the orbital and medial prefrontal cortex of rats, monkeys and humans. *Cerebral cortex* **10**, 206-219 (2000).

12 Slotnick, S. D., Moo, L. R., Segal, J. B. & Hart, J., Jr. Distinct prefrontal cortex activity associated with item memory and source memory for visual shapes. *Brain research. Cognitive brain research* **17**, 75-82 (2003).
